# MicroRNA-99a is a Potential Target for Regulating Hypothalamic Synaptic Plasticity in the Peri/Postmenopausal Depression Model

**DOI:** 10.3390/cells8091081

**Published:** 2019-09-13

**Authors:** Jin Yang, Ling Zhang, Lu-Lu Cao, Jun Qi, Ping Li, Xi-Peng Wang, Xiu-Lan Sun

**Affiliations:** 1 Neuroprotective Drug Discovery Key Laboratory of Nanjing Medical University, Jiangsu Key Laboratory of Neurodegeneration, Nanjing Medical University, Nanjing 211166, China; yj_19950723@outlook.com (J.Y.); zlnjmu@126.com (L.Z.); Nicole_beatific@163.com (L.-L.C.); pli@njmu.edu.cn (P.L.); wxp15851859227@163.com (X.-P.W.); 2 Jiangsu Vocational College of Nursing, Huaian 223001, China; neuropha@njmu.edu.cn; 3 Center for Global Health, School of Public Health, Nanjing Medical University, Nanjing 211166, China

**Keywords:** peri/postmenopausal depression, miR-99a, FKBP51, progesterone receptor, hypothalamic synaptic plasticity

## Abstract

Accumulating evidence has demonstrated that there is a growing trend of menopausal women suffering from depression. However, the pathogenesis of menopausal depression still remains unclear. Hence, this paper aims to reveal the pathological mechanisms involved in postmenopausal depression by using a novel peri- to postmenopausal depression model induced by a two-step ovariectomy plus chronic mild stress (CMS). The results of metabolic chambers and serum hormone/cytokine determination revealed that peri/postmenopausal depressive mice exhibited endocrine and metabolic disorders. Electrophysiological recordings indicated that the hippocampal synaptic transmission was compromised. Compared to the sham group, the microRNA-99a (miR-99a) level decreased significantly in the hypothalamus, and its target FK506-binding protein 51 (FKBP51) enormously increased; in contrast, the nuclear translocation of the progesterone receptor (PR) decreased in hypothalamic paraventricular nucleus (PVN) in the peri/postmenopausal depression mouse model. Additionally, synaptic proteins, including postsynaptic density protein 95 (PSD-95) and synaptophysin (SYN), showed a similar decrease in the hypothalamus. Accordingly, the present work suggests that miR-99a may be involved in the regulation of hypothalamic synaptic plasticity and that it might be a potential therapeutic target for peri/postmenopausal depression.

## 1. Introduction

Depression is a chronical emotional disorder characterized by complicated pathophysiological and neuroendocrine alterations. For women, a decrease in estrogen level during periods of hormone fluctuation, including premenstrual, postpartum, and peri- to postmenopausal periods, is linked to the development and pathological progress of depression [1]. Therefore, beginning during adolescence and throughout women’s lives, women are twice as likely to suffer from depression than men, and they are especially at high risk during their menopause transition [2]. During this period, women undergo rapid hormonal changes, which may also affect the activity of neurons in the brain, leading to mood swings and even depression [3,4]. After decades of research, menopausal depression is still poorly understood and is a controversial topic [5,6]. Hormone treatment, such as estrogen replacement therapy [7], only relieves symptoms in some patients: estrogen administered along with antidepressants [8] could give rise to detrimental effects [9,10]. Due to the lack of clarity in the current forms of treatment, there is an urgent need to determine the underlying pathogenesis of menopausal depression and to identify new therapeutic drugs.

There is a strong association between depression and metabolic syndrome. The nervous and endocrine systems orchestrate adaptive responses to stressful stimuli involving behavioral, emotional, and metabolic alterations [11]. Metabolic disorders, such as obesity and diabetes, are associated with elevated rates of depression [12]. Depression is often associated with increased hypothalamic–pituitary–adrenal (HPA) axis activity and increased levels of glucocorticoids. In particular, increased levels of circulating proinflammatory cytokines and concomitant inflammation activation such as interleukin-6 (IL-6), tumor necrosis factor-α (TNF-α), interleukin-1β (IL-1β) in the brain and blood can lead to depressive symptoms. Several studies have revealed that repeated environmental or psychosocial stress gives rise to synaptic plasticity deficits and neurotransmitter dysregulation, resulting in depressive-like behavior [13].

Synaptic plasticity, one of the most fundamental brain functions, has recently been found to be an indispensable target in treating depression [14]. Mounting evidence has suggested that stress has profound effects on synaptic forms and functions [15]. Synaptic plasticity includes both structure and transmission plasticity. Studies have indicated that postsynaptic density (PSD) molecules, such as PSD-95, are major players in the regulation of synaptic plasticity processes. The modulation of PSD molecules could become a therapeutic strategy in psychiatric diseases [16]. Therefore, we observed synaptic structure, proteins, and postsynaptic density alterations in our study. The effects of stress on synaptic structural and functional plasticity have been particularly well-studied in the hippocampus [14,17,18], whereas they have been studied less in the hypothalamus. Recently, concentration has turned toward understanding and investigating synaptic function and plasticity in the PVN [19], the advanced center of integrating and processing hormones. Thus, we focused on hypothalamic regulation in our research on peri/postmenopausal depression.

MicroRNAs (miRNAs), small noncoding single-stranded RNAs, are critical in the modulation of depression and stress-related diseases [20,21,22]. An increasing number of studies have revealed that miRNAs play a significant role in the central nervous system and are certainly relevant to the structure and function of neurons, the modulation of synaptic plasticity, and transmission, participating in the occurrence and development of depression [23,24,25,26,27,28]. However, miRNA-mediated regulation with respect to peri/postmenopausal depression remains unknown.

Therefore, this paper aims to investigate the pathological phenotypes involved in postmenopausal depression by using a novel peri- to postmenopausal depression model induced by a two-step ovariectomy plus chronic mild stress (CMS), which was established in our previous work [29]. In our study, we identified that peri/postmenopausal depressive mice presented with serious damage in synaptic plasticity, and the decrease in miR-99a might contribute to the dysfunction of synaptic plasticity in peri/postmenopausal depression.

## 2. Materials and Methods

### 2.1. Animals and Model Established as Described Previously 

Seven to eight-week-old female C57BL/6 mice weighing 20 ± 2 g were obtained from the Animal Resource Center of the Faculty of Medicine of Nanjing Medical University for use in the experiment. Stressed group mice were maintained in small individual cages before and during unpredictable chronic mild stress states in small individual cages (29 cm × 18 cm × 16 cm), and nonstressed group mice were housed at 4–5 mice per cage. All mice were kept in a standardized environment (temperature 25 ± 2 °C, humidity 55% ± 10%, a reversed 12-h light/dark cycle (lights on from 20:00 to 08:00)) and housed in the Animal Resource Center of the Faculty of Medicine, Nanjing Medical University. All of the experimental mice were allowed to adapt to the new environment for 1 week before the experiment and were handled 1–2 min per day before surgeries and behavior tests. In this paper, we chose the sham group, and the ovaries were removed in the first and second month with exposure to the chronic mild stress group (CMS + unilateral ovariectomy (UO) + UO) for pathological investigations [29]. The entire experimental process and animal treatment adhered to the rules of the Experimental Animal Application Criteria and approved by Institutional Animal Care and Use Committee (IACUC) of Nanjing Medical University in April 3, 2018. The Approval No. IACUC-1804008.

### 2.2. Metabolic Chambers

After 3 months of model establishment, the peri/postmenopausal depression model (CMS+UO+UO) mice (*n* = 4) and the sham mice (*n* = 4) were placed into a mouse metabolic cage (TSE-PhenoMaster animal metabolic measurement and analysis system) separately for 72 h with a 12-h circadian cycle. Per the system manual, the two groups of mice were placed in the metabolic cage of the metabolic measurement and analysis system. The metabolism of each group of mice was formally recorded after 24 h of adaptive feeding and the following 48-h period. Weight, feed, drinking, total activity, carbon dioxide production (VC0_2_), oxygen consumption (V0_2_), heat production, and blood glucose were assayed. 

### 2.3. Enzyme-Linked Immunosorbent Assay (ELISA)

Serum samples were obtained through a retro-orbital bleeding assay and then clotted overnight at 4 °C before centrifugation for 20 min at 1000× *g*. The concentration of inflammatory cytokines in the serum, including IL-1β, TNF-α, and TGF-β, and endocrine hormones such as corticosterone, T3, progestin, aldosterone, and insulin were detected by an ELISA kit (Cloud Clone Corp., Wuhan, China) according to the manufacturer’s instructions.

### 2.4. Electrophysiological Analysis 

#### Hippocampal Slice Preparations

The mice were anesthetized with isoflurane. The brains were placed in ice-cold artificial cerebrospinal fluid (ACSF) (in mM: NaCl 126, CaCl_2_ 1, KCl 2.5, MgCl_2_ 1, NaHCO_3_ 26, KH_2_PO_4_ 1.25, D-glucose 20, pH 7.4) oxygenated using a gaseous mixture of 95% O_2_ to 5% CO_2_. Coronal slices (400 μm) were cut by a vibrating microtome (Microslicer DTK 1500, Dousaka EM Co., Kyoto, Japan) and perfused in ice-cold cutting solution (in mM: sucrose 94, NaCl 30, KCl 4.5, MgCl_2_ 1.0, NaHCO_3_ 26, NaH_2_PO_4_ 1.2, D-glucose 10, pH 7.4) at 31–34 °C using an in-line heating device (Warner Instruments, Hamden, CT, USA) for 1 h. The slices were transferred to a recording chamber and perfused continually with oxygenated ACSF at a temperature of 30 ± 1 °C.

### 2.5. Field Potential Recording

Orthodromic stimuli were delivered using a bipolar tungsten-stimulating electrode to stimulate Schaffer collateral-commissural fibers using a stimulator (SEN-3301, Nihon Kohden, Tokyo, Japan). Stimulus pulses were delivered every 15 s. Excitatory postsynaptic potential (EPSP) was recorded from the CA1 radiatum layer by a glass microelectrode with 4–5 MΩ resistance that was filled with 2M NaCl. Signals were amplified using an Axoclamp 2B amplifier (Axon Instruments, Foster City, CA, USA), sampled at 20 kHz, and filtered at 10 kHz, and the output was digitized with a Digidata 1200 converter (Axon Instruments). A baseline was established by delivering single pulses (4/min, 0.1 ms pulse width) for 15 min. An input/output (I/O) curve of EPSP slopes was produced by testing stimulation (0.1–1.1 mA) using the software pCLAMP 10.0 (Axon Instrument Inc., CA, USA). Paired-pulse facilitation (PPF) was induced by double stimuli with a 50-ms interpulse interval (IPI). After acquisition of a stable baseline, long term potentiation (LTP) was induced with high-frequency stimuli (HFS, 100 Hz) at the same intensity as baseline test pulses. After delivering HFS, the EPSP slopes were recorded for a further period of 60 min or 180 min. Analyses were made from the percent change of the EPSP slope from baseline 0–5 min after tetanus and 55–60 min or 175–180 min after tetanus. If the EPSP slopes were larger than baseline by 20%, the induction of LTP was determined.

### 2.6. Transmission Electron Microscope 

Mice were anesthetized with isofluorane and perfused with PBS followed by 4% paraformaldehyde (PFA). Brain craniums were removed and hypothalamus samples were cut and fixed with 2.5% glutaraldehyde overnight at 4 °C and then fixed with 1% OsO4 at room temperature for 2 h. After gradient elution by acetone, samples were sliced into ultrathin sections. The samples were subsequently infiltrated in a 1:1 mixture of acetone and Epon resin for 2 h. Ultrathin sections (about 60 nm) were cut on an Ultramicrotome, placed onto copper grids, stained with uranyl acetate and lead citrate, and then observed with a transmission electron microscope.

### 2.7. Immunofluorescence

Mice were anesthetized with isofluorane and perfused with PBS followed by 4% paraformaldehyde (PFA). After postfixation with 4% PFA overnight, the brains were dehydrated using a graded series of alcohol, cleared in xylene, and embedded in paraffin. Then, coronal slices (40 μm) were cut by a rotary microtome (LEICA, RM2245, Heidelberg, Germany) for the hypothalamic paraventricular nucleus (PVN). After deparaffinization, sections of the brain were incubated in 3% H_2_O_2_ for 15 min to block endogenous peroxidases for antigen retrieval and then washed with phosphate-buffered solution (PBS). Next, sections were incubated with 5% goat serum and 0.01% Triton-100 (dissolved in PBS) for 1 h and then stained with primary antibodies (PSD95, 1:400, abcam, Cat# ab12093, Cambridge, UK; FKBP51, 1:200, Proteintech, Chicago, IL, USA) overnight at 4 °C. Following washing with PBS, the sections were incubated with Alexa Fluor 488 or 555 donkey antimouse, antigoat, or antirabbit secondary antibodies (1:1000 dilution, Invitrogen Life Technologies, NY, USA). Images were captured by a fluorescence microscope (Zeiss, Heidelberg, Germany) after incubation with 4-6-diamidino-2-phenylindole (DAPI) for 15 min and washing with PBS.

### 2.8. Nuclear Protein Extraction

Nuclear protein was harvested using a Nuclear and Cytoplasmic Protein Extraction Kit (KGP150/KGP1100, KeyGEN, Nanjing, Jiangsu Province, China). Tissue samples were cut into small pieces, and 900 μL of Buffer A and 100 μL of Buffer B (17 μL of 100-mM phenylmethylsulfonyl fluoride (PMSF) and 1 μL of protease inhibitor/1 mL Buffer A) were added to 50-mg tissue samples, mixed, and homogenized on ice, standing for 30 min. Then, the mixture was centrifuged at 4 °C at 3000 rpm for 10 min. Supernatant was collected into the new centrifuge tube as cytoplasmic protein, and 200 μL of precooled Buffer C (17 μL of 100-mM PMSF and 1 μL of protease inhibitor for every 1 mL of Buffer C before use) was added to the centrifuge precipitate (nucleus), oscillated at the maximum rotational speed violently for 15 s, set to stand for 30–60 min on the ice (oscillating violently for 15 s in 10-min intervals), and centrifuged at 4 °C and 14,000× *g* for 30 min. The supernatant was transferred into a precooling clean centrifuge tube as soon as possible as nuclear protein. Cytoplasmic and nuclear protein concentrations were quantified by a BCA assay kit (Beyotime Biotechnology, Shanghai, China).

### 2.9. Western Blotting

Hypothalamic tissues and cell samples were dissociated in 200 μL of lysis buffer consisting of 1% phenylmethanesulfonyl fluoride (PMSF) (KGP610, KeyGEN, Nanjing, China), and the protein concentration was quantified by a BCA assay kit (Beyotime Biotech Inc., Shanghai, China). The proteins were separated by sodium dodecyl sulfate polyacrylamide gel electrophoresis (SDS-PAGE) and transferred to polyvinylidene fluoride (PVDF) membranes (Roche, Mannheim, Germany). Five percent skim milk in Tris Buffered Saline Tween-20 (TBST) was used to block the membranes for 1 h at room temperature. Following this, the membranes were incubated with the following primary antibodies at 4 °C overnight: PSD95 (1:1000, abcam, Cat# ab12093, Cambridge, UK), Synaptophysin (1:1000, Proteintech, Chicago, IL, USA), FKBP51 (1:1000, Proteintech, Chicago, IL, USA), Lamin B1 (1:1000, Proteintech, Chicago, IL, USA), Progesterone Receptor (1:1000, Bioworld, St. Louis, MO, USA), and Glyceraldehyde-3-phosphate dehydrogenase (GAPDH) (1:5000, Proteintech, Chicago, IL, USA). After washing with TBST, the membranes were incubated with appropriate horse radish peroxidase (HRP)-conjugated secondary antibodies for 1 h at room temperature. After being washed in TBST 4 times, protein bands were detected by enhanced chemiluminescence and Image J software (Version 1.43, NIH, Bethesda, MD, USA).

### 2.10. Chip-Based miRNA Expression Analysis 

Mice hypothalamic tissues were isolated and dissociated in Trizol. The expression level of microRNAs in the hypothalamus of peri/postmenopausal depressive mice (*n* = 3) and the sham mice (*n* = 3) were detected by an ExiqonmiRCURY LNA™ Universal RT microRNA PCR Panel and drawn into a hierarchical cluster graph (Aksomics).

### 2.11. Quantitative Reverse Transcription-Polymerase Chain Reaction (qRT-PCR)

Total RNA was extracted using a TRIzol reagent (Invitrogen Life Technologies, CA, USA) and prepared for quantitative reverse transcription-PCR by using MasterMix (TaKaRa, Japan). Reverse transcription was performed using a MicroRNA Reverse Transcription Kit (GeneCopoeia, USA). Total RNA (800 ng) was reverse-transcribed with 4 μL of 5× PAP/RT Buffer, 0.8 μL of RTase Mix, 0.8 μL of 2.5 U/μL Poly A Polymerase, and RNasefree water added to 20 μL. A reverse transcription reaction was performed in a thermal cycler as follows: 37 °C for 60 min, 85 °C for 5 min, and held at 4 °C. Real-time PCR was carried out using SYBR Green mixture (TaKaRa, Japan) in a QuantStudio 5 system (Thermo Fisher Scientific, New York, USA). The cycling conditions were as follows: denaturation at 95 °C for 30 s, followed by 40 cycles of DNA synthesis at 95 °C for 5 s and 60 °C for 34 s. U6 was used as an endogenous control, and the relative expression of target genes was determined using the 2^−ΔΔct^ method. All-in-One™miRNA Universal Adaptor PCR Primer (GeneCopoeia, Cat NO: QP029) was used as the reverse primer sequence of U70 and miR-99a. The forward primer sequence of U70 used an All-in-One™miRNA qPCR Primer (GeneCopoeia, Cat NO: MmiRQP9022), and the forward primer sequence of miR-99a used the All-in-One™miRNA qPCR Primer (GeneCopoeia, Cat NO: MmiROP0854).

### 2.12. Statistical Analysis

Statistical analyses were performed using SPSS software, version 18.0 (SPSS Inc., Chicago, IL, USA). Multiple comparisons were conducted using one-way ANOVA and Tukey’s multiple comparisons test. The means of the two treatment groups were analyzed using unpaired or paired Student’s *t*-tests. Data are expressed as means ± standard error of measurements (SEMs). A value of p < 0.05 indicates that the difference was statistically significant.

## 3. Results

### 3.1. Peri/Postmenopausal Depressive Mice Had Endocrine and Metabolic Disorders and Synaptic Transmission Compromise

**Figure 1:**
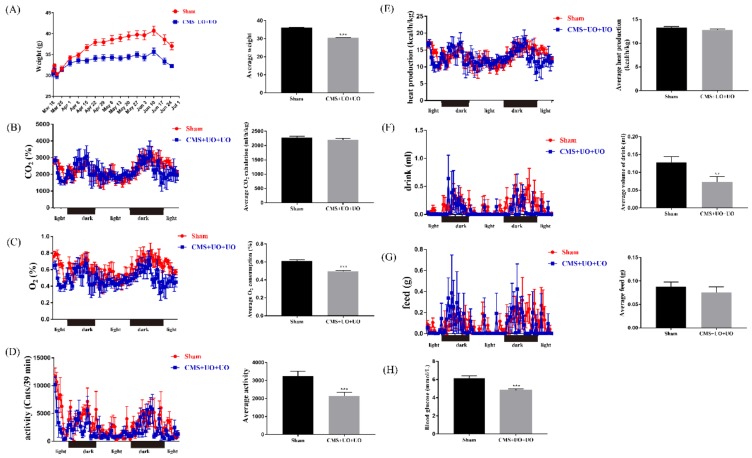
Peri/postmenopausal depression disturbed neuroendocrine and metabolic activity. The insets on the left show the changing curves of weight (**A**), carbon dioxide production (**B**), oxygen consumption (**C**), physical activity (**D**), heat production (**E**), drinking (**F**), and feed (**G**) overtime between the sham group and the model group (CMS+UO+UO) across 24 h. The insets on the right of every section show the quantifications of the total alterations. (**H**) The detection of blood glucose in the two groups.

For comprehensive evaluation of the peri/postmenopausal mouse model, metabolic chambers were applied to assess the metabolic situation in the whole body. As shown in Figure 1A, peri/postmenopausal depressive mice showed a gradual decrease in their weight. Here we found that physical activity and drinking decreased in this model (Figure 1D,F), with no significant differences in feed and heat production (Figure 1E,G). In addition, oxygen consumption of the model mice declined (Figure 1C), and carbon dioxide production (Figure 1B) had no distinction compared to sham mice, which were the essential indicator to reflect energy metabolism. Additionally, we assessed the blood glucose level in the two groups, and we found that the model mice showed a lower blood glucose level (Figure 1H).

**Figure 2:**
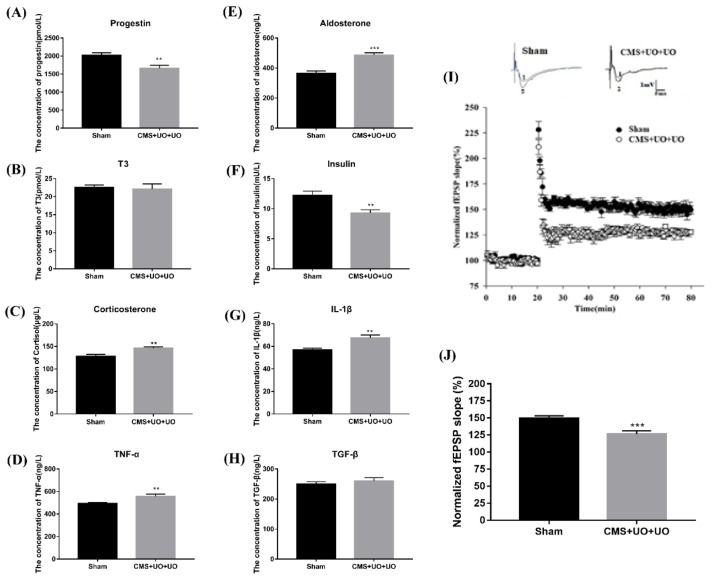
Peri/postmenopausal depression existed in an autonomic nervous dysfunctional and chronic inflammatory status and with synaptic transmission injury. (**A**–**H**) The expression of hormones and cytokines, including progestin, aldosterone, T3, insulin, S-100, IL-1β, TNF-α, and TGF-β in the blood, detected by ELISA. Data are presented as means ± SEMs, *n* = 4~5, ** *p* < 0.01, *** *p* < 0.001 versus sham. (**I**,**J**) Hippocampal synaptic LTP induced by high-frequency stimulation at 100 Hz was recorded in the sham group and in the CMS+UO+UO group.

Since menopause brings about enormous hormone fluctuations, we compared the level of some hormones and cytokine alterations in the serum from the two groups. In addition to the previously detected decline in the estrogen level, we also discovered that the level of progestin decreased by 18% in model mice (Figure 2A). Meanwhile, the levels of corticosterone (Figure 2C) and aldosterone (Figure 2E) minimally increased by 34% and 16%, respectively, while the insulin level (Figure 2F) decreased by 25% and the thyroid hormone (T3) showed no significant change (Figure 2B). Some studies have indicated that insulin resistance and the disruption of these signaling pathways could contribute to psychiatric illnesses [30].

Furthermore, levels of some inflammatory cytokines, including TNF-α (Figure 2D) and IL-1β (Figure 2G), increased by 18% and 13%, and the level of transforming growth factor (TGF-β) (Figure 2G) showed no obvious variation. Previous studies have shown that hippocampal neurotransmitter metabolic disorders also occur in model mice [29]. These results above demonstrated that the peri/postmenopausal depression model presented with endocrine and metabolic disorders, which indicated that peri/postmenopausal depression existed in an autonomic nervous dysfunctional and chronic inflammatory state.

Having determined that peri/postmenopausal depression was linked to metabolism and neuroendocrine dysfunction, we turned our focus toward synaptic plasticity, one of the fundamental pathogeneses of depression. The basal properties of Schaffer collateral-CA1 synaptic transmission were analyzed by plotting field EPSP (fEPSP) slopes against stimulation intensities. Synaptic LTP was induced by high-frequency stimulation at 100 Hz. As expected, the results indicated that peri/postmenopausal depression impaired hippocampal long term potentiation (LTP) induction (Figure 2I,J), meaning synaptic transmission was diminished in peri/postmenopausal depression.

### 3.2. Downregulation of miR-99a in the Hypothalamus of Peri/Postmenopausal Depressive Mice

**Figure 3:**
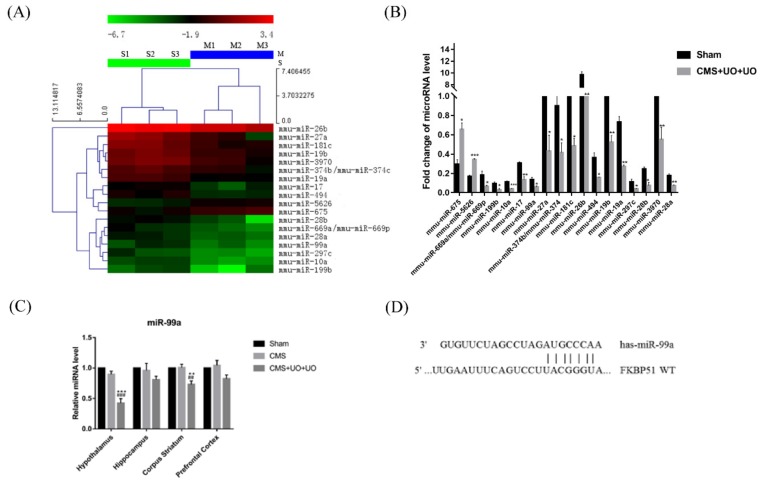
The expression level of miR-99a was downregulated in the hypothalamus of peri/postmenopausal depressive mice. (**A**) The microRNA (miRNA) expression profile analyzed by a miRNA chip between the sham group and the CMS+UO+UO model. (**B**) Quantification of miRNAs in the two groups screened from the chip. (**C**) The miR-99a expression levels in the hypothalamus, hippocampus, corpus striatum, and prefrontal cortex were detected by quantitative reverse transcription-polymerase chain reaction (qRT-PCR). (**D**) MiR-99a bonds to the 3′UTR of FKBP51. Data are presented as means ± SEMs, *n* = 4–5, * *p* < 0.05, ** *p* < 0.01, *** *p* < 0.001 versus sham.

We next sought to investigate the underlying mechanisms of pathological alterations in peri/postmenopausal depressive mice. Since the hypothalamus is the advanced center of integration and processing of hormones, our attention was focused on hypothalamic miRNA regulation in our peri/postmenopausal depression research. Based on the chip-based miRNA expression analysis, we found the expression of 18 microRNAs altered in the hypothalamus of CMS+UO+UO mice (Figure 3A,B). One study indicated that miR-99a could be linked to long-term estrogen deprivation [31]. One clinical study indicated that progesterone and estrogen were negatively correlated with the expression level of miR-99a [32]. Thus, we speculated that miR-99a could be related to menopausal depression.

Specifically, we detected miR-99a expression in the hypothalamus among the sham, CMS, and CMS+UO+UO groups. The results indicated that miR-99a was dramatically downregulated in the hypothalamus of peri/postmenopausal depression model mice. The corpus striatum showed mild changes, and the CMS model suggested no significant difference (Figure 3C). Accordingly, we made an assumption that hypothalamic miR-99a could have a close correlation with peri/postmenopausal depression. 

### 3.3. Hypothalamic FKBP51 Was Upregulated and Progesterone (PR) Nuclear Translocation Decreased in the Peri/Postmenopausal Depression Mice

**Figure 4:**
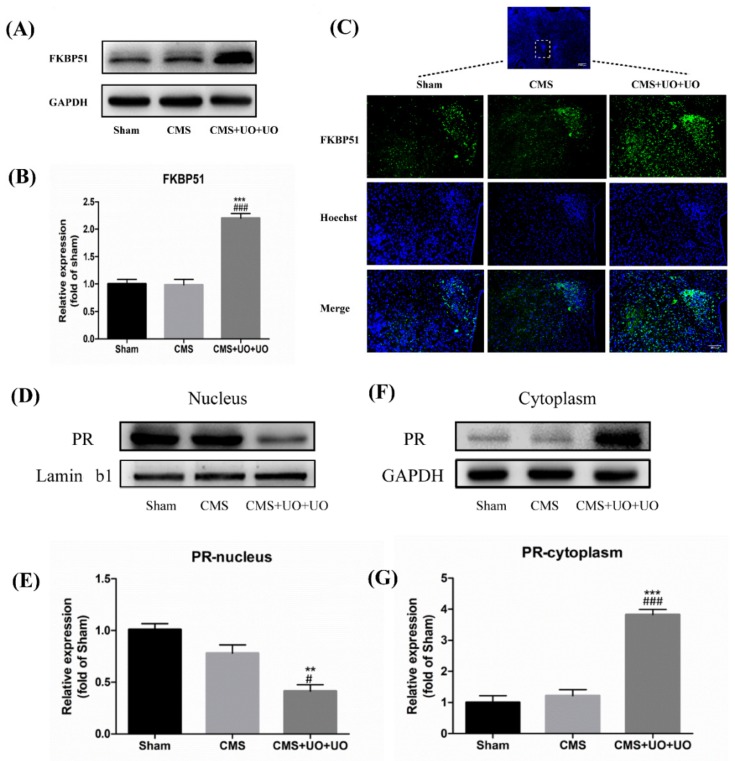
Hypothalamic progesterone (PR) nuclear translocation decreased and FKBP51 was upregulated in peri/postmenopausal depression. (**A**–**C**) The expression of FKBP51 in the hypothalamus was detected by western blot and immunofluorescence. (**D**–**G**) The expression of PR in the nucleus and cytoplasm of the hypothalamus was detected by western blot using Lamin b1 and GAPDH as loading controls. Data are presented as means ± SEMs, *n* = 4–5, ** *p* < 0.01, *** *p* < 0.001 versus sham; ^#^ *p* < 0.05, ^###^ *p* < 0.001 versus CMS group.

The TargetScan algorithm showed that miR-99a could target the 3′UTR of FKBP51 and thus negatively regulate its expression (Figure 3D), which has been demonstrated by a dual luciferase reporter assay in other studies [33]. Consistent with these investigations, we found that the level of FKBP51 sharply increased in the hypothalamus of CMS+UO+UO model mice compared to the sham group, while the CMS group did not show any significant difference when compared to the sham group (Figure 4A–C). Several studies have indicated that FKBP51 determines the cytoplasmic localization of PR [34]. Next, we detected the PVN PR nuclear translocation in the sham group, CMS model group, and CMS+UO+UO group. In contrast to the sham group, the CMS+UO+UO group showed that the PVN PR nuclear translocation was obviously restrained, while the CMS group showed no significant difference when compared to the sham group (Figure 4D–G). These results suggest that the high expression of FKBP51 could inhibit PR nuclear translocation in the hypothalamus, thus affecting PR function.

### 3.4. Hypothalamic Synaptic Plasticity Injury Was More Severe in the Peri/Postmenopausal Depressive Mice

**Figure 5:**
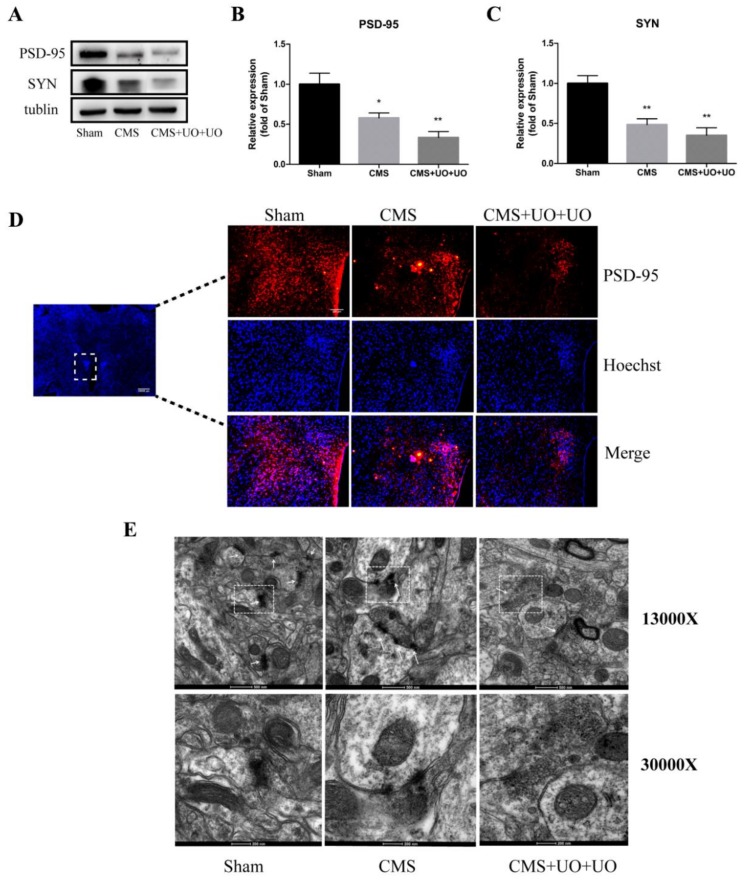
Hypothalamic synaptic plasticity injury in peri/postmenopausal depressive model mice. (**A**–**D**) The expression of synaptic proteins (PSD-95) and synaptophysin in the PVN in the sham, CMS, and CMS+UO+UO groups was detected by western blot and Immunofluorescence (IF). (**E**) The morphologies of synaptic structure in the hypothalamus in the sham, CMS, and CMS+UO+UO groups were detected by transmission electron microscope. Data are presented as means ± SEMs, *n* = 4–5, * *p* < 0.05, ** *p* < 0.01 versus sham.

Studies have indicated that PR is associated with multiple proteins, including nuclear receptor coactivators and synaptic proteins, thus modulating synaptic plasticity [35]. Synaptic plasticity injury is not a unique occurrence in peri/postmenopausal depression, as it occurs in most common instances of depression. On the basis of this finding, we compared the differences in synaptic plasticity alterations between the sham group, CMS model group, and CMS+UO+UO group. We found that the peri/postmenopausal depression mouse model showed a sharper decline in levels of synaptic proteins (PSD-95) and synaptophysin in the PVN of the hypothalamus compared to the CMS model (Figure 5A–D). The electron microscope results indicated that the synaptic structure was damaged and the postsynaptic density attenuated in the hypothalamus of the peri/postmenopausal depressive model mice, manifesting more severe injury in the peri/postmenopausal depressive model mice than in the CMS model mice (Figure 5E). Altogether, these data show that the peri/postmenopausal depressive model mice suffered more severe hypothalamic synaptic plasticity injury.

## 4. Discussion

The odds of depressive symptoms in the perimenopause period are doubled compared to premenopause and postmenopause [36]. Our study focused on the occurrence of depression during menopausal transition rather than simply focusing on the premenopause or postmenopause period. Our findings demonstrate that the pathogenesis of peri/postmenopausal depression in hormonal regulation is more complicated and sophisticated than in common depression.

Menopausal transition is usually accompanied by hormonal fluctuations, dysfunction of the HPA axis, and abnormal metabolism, each of which influences women’s health both physiologically and mentally and may finally lead to various diseases [37]. In addition to estrogen and progestin waves, some studies have found an increase in cortisol levels from the early to late menopausal transition stage, indicating that women in this stage are more vulnerable to stress. In accordance with these clinical manifestations, we also detected that levels of stress-related hormones, such as corticosterone and aldosterone, increased, and endocrine hormones such as estrogen, progestin, and insulin showed an inverse decrease in the peri/postmenopausal depression model. Clinical studies have revealed that circulating markers of immune activation, such as increased numbers of granulocytes and monocytes and elevated levels of TNF-α and IL-6, are observed in the blood of individuals with depression [13]. TNF-α, IL-6, and IL-1β in the serum of the model mice showed a similar increase. In our research, we also observed that depression was a disease that caused dysfunction in the metabolism of the entire body. These results, together, reveal that the neuroendocrine system and metabolism in peri/postmenopausal depression are in a chaotic state and that the brain is in a constant inflammatory state.

An elegant study revealed that menopause disrupts estrogen homeostasis, which decreases the stimulation of ERβ-mediated estrogen signaling, thus leading to decreased levels of brain-derived neurotrophic factor (BDNF) and reduced BDNF/TrkB signaling. Consequently, this weakens hippocampal synaptic strength, thus compromising the brain’s ability to adapt and increasing the risk of depression [38]. Our previous study also observed that ER-β was downregulated [29] and that hippocampal synaptic transmission was diminished in a peri/postmenopausal depression model. 

Based on the chip-based miRNA expression analysis and the results of the RT-qPCR, we found that miR-99a decreased and FKBP51 sharply increased in the peri/postmenopausal depressive mice compared to the sham mice, and there was no difference in the CMS mice. Some studies have shown that the absence of FKBP51 could increase the localization of PR in the nucleus [34]. Accordingly, we found that PR nuclear translocation was severely hindered in the hypothalamus of peri/postmenopausal model mice in contrast to sham mice, while no significant difference was noted in the CMS group. On this basis, PR could be involved in regulating synaptic proteins, thus modulating synaptic plasticity in the hypothalamus [35]. Synaptic plasticity is the ability to sense, assess, and store complex information and to make appropriate, adaptive responses to subsequent related stimuli, the mechanisms of which have been linked to the pathophysiology and treatment of multiple neurobiological disorders, including depression [12]. The present data suggest that peri/postmenopausal depression displayed more severe injury to synaptic structure and synaptic protein downregulation in the PVN of the hypothalamus.

Therefore, we suggest that miR-99a/FKBP51/PR might be responsible for manipulating and regulating hypothalamic synaptic plasticity in peri/postmenopausal depression, which might be a potential therapeutic target for peri/postmenopausal depression. Further studies should observe the actions of miR-99a in peri/postmenopausal depression and confirm the effects of miR-99a in menopausal women suffering from depression in order to develop it as a therapeutic target for menopausal depression in the future.
